# Copy number variation of microRNA genes in the human genome

**DOI:** 10.1186/1471-2164-12-183

**Published:** 2011-04-12

**Authors:** Malgorzata Marcinkowska, Maciej Szymanski, Wlodzimierz J Krzyzosiak, Piotr Kozlowski

**Affiliations:** 1 Institute of Bioorganic Chemistry, Polish Academy of Sciences, Poznan, Poland; 2 Computational Genomics Laboratory, Institute of Molecular Biology and Biotechnology, Adam Mickiewicz University, Poznan, Poland

## Abstract

**Background:**

MicroRNAs (miRNAs) are important genetic elements that regulate the expression of thousands of human genes. Polymorphisms affecting miRNA biogenesis, dosage and target recognition may represent potentially functional variants. The functional consequences of single nucleotide polymorphisms (SNPs) within critical miRNA sequences and outside of miRNA genes were previously demonstrated using both experimental and computational methods. However, little is known about how copy number variations (CNVs) affect miRNA genes.

**Results:**

In this study, we analyzed the co-localization of all miRNA *loci *with known CNV regions. Using bioinformatic tools we identified and validated 209 copy number variable miRNA genes (CNV-miRNAs) in CNV regions deposited in Database of Genomic Variations (DGV) and 11 CNV-miRNAs in two sets of CNVs defined as highly polymorphic. We propose potential mechanisms of CNV-mediated variation of functional copies of miRNAs (dosage) for different types of CNVs overlapping miRNA genes. We also showed that, consistent with their essential biological functions, miRNA *loci *are underrepresented in highly polymorphic and well-validated CNV regions.

**Conclusion:**

We postulate that CNV-miRNAs are potential functional variants and should be considered high priority candidate variants in genotype-phenotype association studies.

## Background

MicroRNAs (miRNAs) are a family of short (~20 nt), single-stranded, noncoding RNAs that are primarily involved in post-transcriptional down-regulation of gene expression in most eukaryotes [1]. Specific miRNAs are engaged in a variety of processes, including development, cell proliferation, differentiation and apoptosis [2]. Numerous studies have demonstrated that aberrant over-expression or down-regulation of certain miRNAs contribute to carcinogenesis and that these miRNAs can therefore be classified as either oncogenes (oncomirs) or tumor suppressors, respectively [3].

Mature, functional miRNAs are generated from primary precursors (pri-miRNA) encoded either by independent transcriptional units or within protein- or RNA-coding genes. In mammals, maturation of miRNAs involves two subsequent RNA cleavage steps. The first step takes place in the nucleus and is carried out by the Drosha nuclease to produce the secondary precursor (pre-miRNA) [4]. The pre-miRNAs (~60 nt) possess a hairpin structure, with the double-stranded portion interrupted by one or more mismatched nucleotides. Upon export to the cytoplasm, the pre-miRNA is further processed into an miRNA duplex by the RNAse III Dicer; [5] one of the duplex strands (passenger) is released, and the other serves as the mature miRNA [6]. The miRNA-induced silencing complex (miRISC) interacts with complementary target sequences, which are usually located within the 3' untranslated regions (3'UTRs) of mRNAs, causing mRNA degradation or inhibition of translation [7-9].

It is estimated that, in humans and other mammals, the expression of at least one-third of protein-coding genes is fine-tuned by approximately 1,000 miRNAs [10,11]. Currently, over 700 human miRNAs have been identified, and their sequences are deposited in miRBase (the microRNA database; http://www.mirbase.org).

Polymorphisms in miRNA genes can affect the expression of many downstream-regulated genes [12,13]. The most common form of polymorphism that affects the function of an miRNA (e.g., the structure of miRNA precursors, the efficiency of miRNA biogenesis and miRNA-target recognition) is the single nucleotide polymorphism (SNP). Computational and experimental studies have revealed many SNPs located in different parts of pre-miRNA sequences [14-16]. The occurrence of SNPs (including INDELs) in pre-miRNA regions is significantly lower than that in the surrounding reference sequences [16]. While sequences of mature miRNAs are the most conserved, the sequences of anti-miRNAs and the stems (outside miRNA and anti-miRNA) and loops of pre-miRNAs are somewhat less conserved [16]. SNPs naturally occurring within pre-miRNA sequences may affect miRNA biogenesis and impair miRNA-mediated gene silencing, as demonstrated by functional assays [15,17]. Recently, large genome-wide association study has demonstrated that also SNPs located outside (>14 kb) of pre-miRNA sequences can modulate miRNA expression both as *cis- *and *trans-*regulators (miRNA-eQTLs). One of identified miRNA-eQTLs (rs1522653) was shown to correlate with expression of 5 different miRNAs [18].

MiRNA target sites are also conserved genetic elements. Bioinformatic analyses show that SNPs are underrepresented in both experimentally validated and computationally predicted miRNA target sites, [16,19] and SNPs have the potential to either disrupt or create new miRNA target sites [19]. It has also been proposed that target site polymorphisms may play a role in evolution by altering miRNA specificity and function.

However, little is known about copy number variation (CNV) of miRNA genes. CNVs are segments of genomic DNA (roughly 1 kb to 1 Mb in length) that show variable numbers of copies in the genome due to deletions or duplications. CNVs recurrently occurring in a population are often called copy number polymorphisms (CNPs). Only a few CNV discovery studies report the presence of miRNAs in detected CNV regions and recognize their potential consequences [20-22]. Indeed, it was suggested that a comprehensive analysis of the co-localization of miRNAs and CNVs is needed [12].

Numerous studies show that CNVs can influence the expression of protein-coding genes in a copy number-dependent manner [23-25]. Recent results of genome-wide association study has confirmed such association for dozens of protein-coding genes and showed that CNVs capture at least 18% of the total detected genetic variation in gene expression [26]. It seems obvious that the expression of miRNA genes can also be modified by CNVs. This notion is supported by results from cancer genetics studies. For instance, there is a correlation between somatic copy number variation and the expression of miRNA genes, and miRNA genes recurrently amplified or lost in cancer genomes can serve as oncogenes or cancer suppressor genes, respectively [27-31].

In this study, by comparing the coordinates of human miRNAs with different sets of CNV regions (DGV-deposited and highly polymorphic), we identified over 200 human copy number variable miRNA *loci*. By comparing fractions of miRNAs and the genome that are covered by differentially validated CNV regions, we showed that miRNA *loci *are underrepresented in highly polymorphic CNVs, but not in CNVs deposited in the DGV database. We discuss the potential functional relevance of identified copy number variable miRNAs and propose models of how different types of CNVs can affect miRNA dosage.

## Results and Discussion

Prior to bioinformatic identification of copy number variable miRNA genes (CNV-miRNAs), we compared the frequency of SNPs in annotated pre-miRNA sequences (3.7 SNPs/1,000 bp) and in reference human genome (4.8 SNPs/1,000 bp). Significantly lower number of SNPs in the pre-miRNA sequences (Fisher's exact test; p < 0.0001) most likely results from SNP purification effect and confirms general conservation of the analyzed pre-miRNA sequences. These analyses confirmed a SNP purification effect in pre-miRNA sequences reported previously [16]. The much higher number of SNPs identified in annotated pre-miRNA sequences in our study (N = 229; Additional file 1) versus N = 65 reported previously [16] results from the increased number of both SNPs (dbSNP - build 130; Apr 30, 2009; only annotated as 'single'; ~14 million SNPs) and miRNAs (miRBase - v 13.0), available in versions of databases used in this study.

To identify CNV-miRNAs, we compared the positions of miRNA *loci *with three sets of CNVs: 'DGV-deposited' (N = 29133; 30% genome coverage), 'polymorphic-SMC' (N = 1319; 1.2% genome coverage) [32] and 'polymorphic-DC' (N = 5037; 2.3% genome coverage) [22] CNVs. 'DGV-deposited' CNVs include all 29133 CNVs deposited in the Database of Genomic Variants (DGV update Aug 05, 2009 - http://projects.tcag.ca/variation). Two sets of 'polymorphic' CNVs ('polymorphic-SMC' [32] and 'polymorphic-DC' [22]) include highly polymorphic CNVs (minor allele frequency >0.01) validated by high-quality genotyping in two recent CNV-discovery studies using CNV-dedicated high-density hybrid arrays (combining traditional SNP probes and probes targeting CNVs) [22,32]. In both of these studies, precise breakpoints and unambiguous copy numbers were determined for each analyzed sample. All 'DGV-deposited' CNV-miRNA regions were further characterized by the following validation factors: (i) number of publications reporting CNVs (references), (ii) number of overlapping CNVs (DGV records) and (iii) number of observations in discovery studies (frequency) (Additional file 2). Since the exact boundaries of miRNA genes (including regulatory elements) are difficult to determine, we used the genomic coordinates of all pre-miRNA *loci *deposited in miRBase (v 13.0; N = 715) as a proxy of miRNA gene sequences (three pre-miRNA *loci *located in the mitochondrial genome were excluded from our analysis) [33,34]. We realize, however, that CNVs overlapping other functional regions of miRNA coding genes (e.g., promoters) can also affect miRNA biogenesis and functionality, and those CNVs will be missed in our analysis.

The CNV-miRNAs identified in 'DGV-deposited' CNVs (N = 209) and in two sets of 'polymorphic' CNVs (N = 4 and N = 8) are shown in Additional file 2 and Table 1, respectively. Top-validated 'DGV-deposited' CNV-miRNAs are also shown in Table 2. Most miRNA *loci *identified in 'polymorphic' CNVs also overlapped with top-validated 'DGV-deposited' CNV regions (Table 1 and Table 2). All 'polymorphic' CNV-miRNAs were relatively frequent (combined minor genotype frequency >0.1 in at least one HapMap population). Among the identified miRNA-CNVs, we found deletions (e.g., hsa-mir-384 and hsa-mir-1324), duplications (e.g., hsa-mir-1972 and hsa-mir-1977), and multiple duplications (multiallelic polymorphisms; e.g., hsa-mir-1233 and hsa-mir-1268). The number of observed copies ranged from 0 (e.g., hsa-mir-384 and hsa-mir-650) to 6 (e.g., hsa-mir-1268).

**Table 1:** miRNA *loci *localized in polymorphic CNV regions

miRNAs localized in 'polymorphic-SMC' CNV regions								
**miRNA ID**	**miRNA position**	**dupl.**	**CNV region position**	**genotypes**	**CNV ID**	**functional relevance**	**expression (mimiRNA/**[18]**)**	**conservation**

mir-1268	chr15:20014593-20014644		chr15:19803370-20089386	2,3,4,5,6	2057	1) recurrently deleted in classical Hodgkin's lymphoma [47]	not reported/NA	primates
mir-1233	chr15:32607783-32607864	chr15	chr15:32487975-32617680	0,1,2,3	2082	1)	not reported/NA	primates
mir-1972	chr16:15011679-15011755	chr16	chr16:14897364-15016088	2,3,4	2141		not reported/NA	primates
mir-384	chrX:76056092-76056179		chrX:76053855-76057477	0,1,2	2648		in several tissues/NA	mammals

**miRNAs localized in 'polymorphic-DC' CNV regions**								

**miRNA ID**	**miRNA position**	**dupl.**	**CNV region position**	**genotypes**	**CNV ID**	**functional relevance**	**expression (mimiRNA/**[18]**)**	**conservation**

mir-1977	chr1:556050-556128	chrM	chr1:554403-560267	2,3,4	3.1		not reported/NA	primates
mir-1324	chr3:75762604-75762699		chr3:75464498-75782745	1,2	1432.2		not reported/NA	primates
mir-548i-2	chr4:9166887-9167035		chr4:9117494-9354801	1,2	1815.3		not reported/NA	primates
mir-1275	chr6:34075727-34075806		chr6:34071086-34077139	1,2	2853.1	2) upregulated in blood cells of MS patients [41]	not reported/NA	primates
mir-1302-2	chr9:20144-20281	chr1, 15,19	chr9:485-38531	2,3	4134_full		not reported/NA	primates
mir-1233	chr15:32461562-32461643	chr15	chr15:32450046-32662643	2,3,4,5	6351.3	1)	not reported/NA	primates
mir-1233	chr15:32607783-32607864	chr15	chr15:32450046-32662643	2,3,4,5	6351.3	1)	not reported/NA	primates
mir-650	chr22:21495270-21495365		chr22:20711019-21578950	0,1,2	8103_full	1)	in several tissues (mostly ovary and ovary-derived cancers)/high	primates

dupl. - localization of duplicated copies; mimiRNA/[18] - miRNA expression according to database mimiRNA/and according to resent result of expression analysis in primary fibroblast cells (high - high expression, absent - low or undetectable expression in fibroblast cells, NA - not analyzed).

**Table 2:** miRNA *loci *localized in CNV regions validated by multiple overlapping CNVs

miRNAs localized in 'DGV-deposited' CNV regions validated by multiple overlapping CNVs							
**miRNA ID**	**miRNA position**	**dupl.**	**minimal CNV region**	**# CNVs**	**functional relevance**	**expression (mimiRNA/**[18]**)**	**conservation**

mir-1977	chr1:556050-556128	chrM	chr1:554340-569354	6		not reported/NA	primates
mir-149	chr2:241044091-241044179		chr2:241039698-241051687	6	3) downregulated in squamous cell carcinoma of the tongue [44]	in multiple tissues/high	vertebrates
mir-566	chr3:50185763-50185856		chr3:50173490-50214015	7		in several tissues/absent	primates
mir-1324	chr3:75762604-75762699		chr3:75761737-75839337	6		not reported/NA	primates
mir-570	chr3:196911452-196911548		chr3:196905807-196918722	9		in several tissues/absent	primates
mir-548i-2	chr4:9166887-9167035		chr4:9152768-9182838	9		not reported/NA	primates
mir-548i-3	chr8:7983873-7984021		chr8:7965981-8024983	14		not reported/NA	primates
mir-383	chr8:14755318-14755390		chr8:14741501-14763659	8	4) downregulated in non-obstructive azoospermia [39]	in multiple tissues/absent	vertebrates
mir-661	chr8:145091347-145091435		chr8:145090343-145104971	8	5) downregulates the expression of metastatic tumor antigen 1 (MTA1), inhibits the motility, invasiveness, anchorage-independent growth, and tumorigenicity of cancer cells [48]	in several tissues (mostly ovary and ovary-derived cancers)/absent	primates
mir-1299	chr9:68292059-68292141		chr9:68291272-68298205	7		not reported/NA	primates
mir-126	chr9:138684875-138684959		chr9:138680837-138688363	14	6) suppresses cell growth in colon cancer [43]; downregulates HOXA9, playing a role in the development of many organs and often upregulated in myeloid leukemias [37]; regulates angiogenic signaling and vascular integrity [38]; overexpressed in ALL and AML [42]	high, in multiple tissues/high	vertebrates
mir-202	chr10:134911006-134911115		chr10:134903011-134918923	10		in several tissues/absent	vertebrates
mir-1268	chr15:20014593-20014644		chr15:19975453-20046356	37	1) see Table 1	not reported/NA	primates
mir-1233	chr15:32461562-32461643	chr15	chr15:32461525-32469857	9	1) see Table 1	not reported/NA	primates
mir-1233	chr15:32607783-32607864	chr15	chr15:32599966-32615283	17	1) see Table 1	not reported/NA	primates
mir-662	chr16:760184-760278		chr16:750040-764098	6		in several tissues/absent	primates
mir-1972	chr16:68621750-68621826	chr11	chr16:68621490-68653097	6		not reported/NA	primates
mir-142	chr17:53763592-53763678		chr17:53751608-53767652	11	7) increased expression correlates with rejection of organ transplants [40]; overexpressed in pre-B-ALL patients [46]; potentially involved in the development of blood cancer or brain tumors [45]	high, in multiple tissues/absent	vertebrates
mir-1270	chr19:20371080-20371162		chr19:20370872-20383238	9		not reported/NA	primates
mir-663	chr20:26136822-26136914		chr20:26136626-26139184	6		in several tissues/NA	primates
mir-650	chr22:21495270-21495365		chr22:21494381-21502189	38	1) see Table 1	in several tissues/high	primates
mir-514-2	chrX:146171153-146171240		chrX:146168796-146174575	6		in several tissues/NA	mammals
mir-514-3	chrX:146173851-146173938		chrX:146168796-146174575	6		in several tissues/NA	mammals

dupl. - localization of duplicated copies; mimiRNA/[18] - miRNA expression according to database mimiRNA/and according to resent result of expression analysis in primary fibroblast cells (high - high expression, absent - low or undetectable expression in fibroblast cells, NA - not analyzed).

The sequences of miRNA deposited in miRBase are derived from discovery studies in which many strict miRNA verification criteria were applied (e.g. hairpin forming potential, evolutionary conservation, presence in multiple clones/sequence reads or homogeneity of the 5'end). The SNP frequency analysis presented in this study also confirmed global conservation of annotated pre-miRNA sequences. However, there is still a possibility that some of the miRNAs in the miRBase represent experimental artifacts of false positive discoveries [35]. To provide additional data that can further validate miRNAs identified in CNVs we have conducted bioinformatic analysis of their expression and conservation. Table 1 and Table 2 show that according to different miRNA expression resources summarized in mimiRNA database [36] over half (14/26) of top-validated CNV-miRNAs (Table 1 and Table 2) were shown to be expressed in at least several tissues/cell lines (detailed expression profiles are shown in Additional file 3). MiRNA whose expression is not reported in mimiRNA were either not analyzed for expression or did not show expression in the analyzed tissues. Additionally, three out of ten (30%) top-validated CNV-miRNAs (Table 1 and Table 2) which expression in primary fibroblast cell lines was analyzed by the micro-fluidics-based TaqMan Human MiRNA Array show high level of expression [18]. Based on the currently available sequence data for miRNAs deposited in miRBase and blast searches of the vertebrate genomic sequences we also determined evolutionary conservation of the miRNAs found in top-validated CNV regions. Most of these miRNAs seem to be specific only for primates. There are, however, 8 miRNAs that are conserved across mammals or vertebrates (Table 1 and Table 2).

The functional relevance of several of the CNV-miRNAs identified in this survey was previously reported in the literature (manual screening; Table 1 and Table 2). CNV-miRNAs are involved in many processes and phenotypes (diseases), including organ development [37], angiogenesis [38], male infertility [39], transplant rejection [40], multiple sclerosis [41] and cancer. Many CNV-miRNAs are specifically deleted, amplified or expressed in different types of cancers [42-47] and can regulate the expression of important cancer-related genes [37,48]. The copy number variation of those functionally relevant miRNAs can modulate or predispose one to the aforementioned phenotypes.

**Figure 1:**
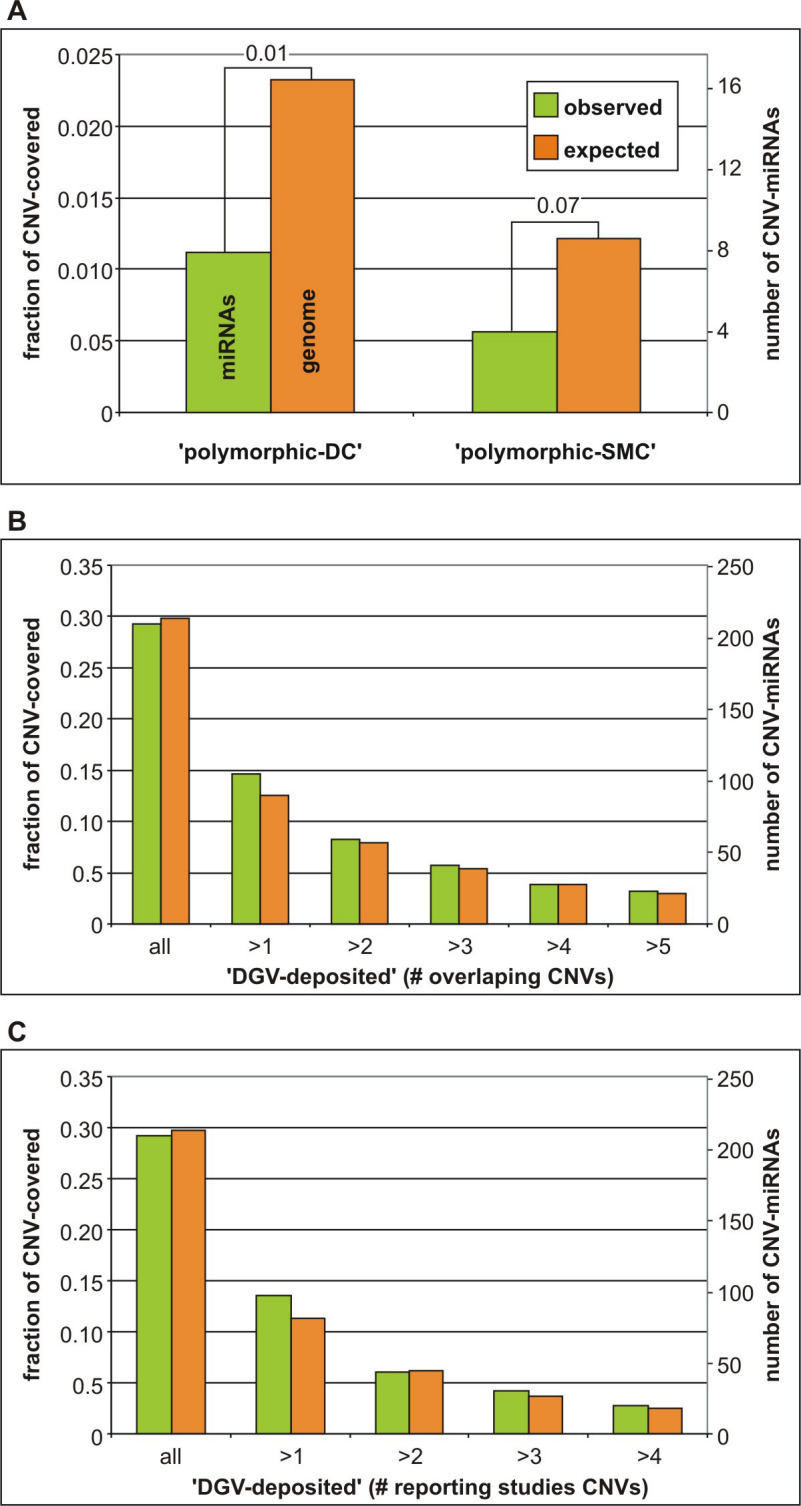
**Comparison of observed and expected number (fraction) of miRNA *loci *located in different CNV regions**. Expected values were estimated based on the fraction of the genome covered by CNVs. A) Graph showing the fractions of miRNA *loci *(observed number of CNV-miRNAs; green bars) and the genome (expected number of CNV-miRNAs; orange bars) covered by two sets of 'polymorphic' CNVs. Binomial probabilities of equal or lower than the observed number of miRNA *loci *covered by CNVs are indicated over the bars. B) and C) The fractions of miRNA *loci *and the genome covered by 'DGV-deposited' CNV regions gradually narrowed by the increasing number of overlapping CNVs (DGV records) (B) and the increasing number of reporting references (C).

In the next step, we determined whether the overlap of CNVs and miRNA *loci *was random (null hypothesis) or whether the CNVs were underrepresented at these *loci *(alternative hypothesis). To test this hypothesis, we compared fractions of miRNA *loci *and fractions of the genome covered by differentially defined CNV regions. Figure 1A shows that the fraction of miRNA *loci *covered by two sets of 'polymorphic' CNVs is approximately two times lower than expected (fraction of the covered genome). Although this effect was only marginally significant (Figure 1A), it suggested that at least highly polymorphic CNVs are under negative (purifying) selection at miRNA genes. Conversely, the fraction of miRNAs (0.292) covered by 'DGV-deposited' CNVs corresponded almost exactly to the fraction of the genome covered by those CNVs (0.299). The CNV purification effect was not observed, even after narrowing 'DGV-deposited' CNV regions by different validation factors defined above (Figure 1B and 1C). The fact that the purifying effect did not apply to the 'DGV-deposited' CNVs suggested that a significant portion of these CNVs are very rare, private, or significantly oversized or represents false positive artifacts. This observation is consistent with the conclusions from other recently published results [32,49].

**Figure 2:**
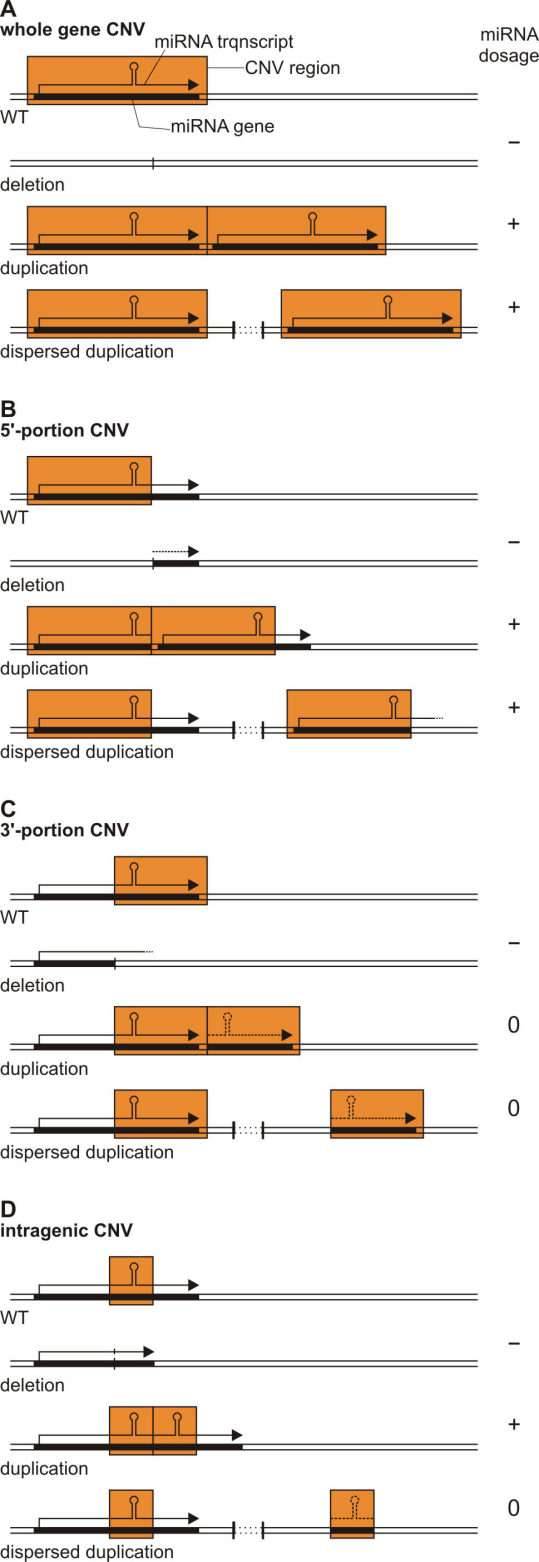
**Potential mechanism of CNV-mediated variation of miRNA dosage**. Schematic representation of an miRNA gene and its primary transcript (solid or dotted arrow-lines). The position of the pre-miRNA sequence is indicated as a hairpin-loop structure in the miRNA primary transcript. Dotted lines represent transcripts unlikely to be produced due to the lack of promoter and transcriptional start sequences. Orange boxes represent CNV regions (deletions, duplications and dispersed duplications). The following panels show a CNV spanning different parts of the miRNA gene: (A) whole gene, (B) 5'-portion, (C) 3'-portion and (D) intragenic region of the gene. +, - and 0 indicate potential increase, decrease and no change of miRNA dosage, respectively.

Although copy number variation can influence gene expression through different mechanisms (e.g., position effect and deletion or duplication of regulatory elements that control transcription or splicing), the most obvious mechanism is in the variability of dosage (number of functional copies). All of these mechanisms can affect both protein-coding and miRNA genes. However, mechanisms of dosage variation may be different for protein-coding and miRNA genes. In Figure 2, potential consequences of different CNV types overlapping different parts of miRNA genes are proposed. Not only whole gene amplification but also certain partial gene duplications (multiple duplications) can increase the dosage of miRNAs. Conversely, partial gene deletions may not always result in decreased miRNA dosage. This contrasts with the situation observed for protein-coding genes, in which only duplication of the entire gene (including the promoter and regulatory sequences) can lead to an increased number of functional copies, and almost every (even partial) gene deletion is deleterious.

Analysis of 11 miRNAs located in CNVs with well defined breakpoints (Table 1) showed that (i) 3 of these miRNAs are located in the protein coding genes which are entirely positioned within CNVs, (ii) 4 of the miRNAs are located in intergenic regions and are flanked by at least 20 kb of CNV sequences, (iii) 3 miRNAs are located in intergenic regions flanked by short CNV sequences (< 5 kb) and (iv) 1 miRNA is located in a gene of which the 3'end extends beyond CNV (Additional file 4). Taking into account the average size of a human gene (~30 kb) one can expect that miRNAs located in large CNVs (groups (i) and (ii)) will be expressed from genes entirely embedded within the CNV regions. According to the model presented in Figure 2A the expression of such miRNAs very likely will correlate with expression (number of copies) of genes from which these miRNAs are generated (no matter whether generated from protein-coding or non-coding transcripts). MiRNA located in short CNVs (group (iii)) most likely will form the tandem copies transcribed from one promoter. A number of such copies may modulate the number of miRNA precursors (pre-miRNAs) present in one primary transcript (pri-miRNA) and thus may modulate expression of miRNA (Figure 2D). Expression of miRNA whose gene only partially is embedded in CNV (iii) may be modified according to the model shown in Figure 2B and will depend on expression and stability of the transcript truncated at the 3'end. Moreover, it should be noted that some pre-miRNA sequences occur in the genome in multiple copies. Although the functionality of such copies is still mostly unknown, the duplicated copies of miRNA genes may mask the effect of copy number variations that usually affect only one copy.

Finally, not only common CNVs, but also CNVs implicated in specific diseases can affect miRNA *loci *and thus can play important role in pathogenesis. We have identified 38 *loci *of miRNAs located in chromosomal regions implicated in microdeletion/microduplication syndromes (DECYPHER v5.0 [50]) (Additional file 5). For example, six miRNA *loci *(hsa-mir-185, hsa-mir-1306, hsa-mir-1286, hsa-mir-649, hsa-mir-301b and hsa-mir-130b) are located within genomic region implicated in DiGeorge syndrome. The role of somatic copy number variation of miRNA genes in cancer is extensively investigated in multiple studies (e.g. [27-31]) and was recently summarized in several review articles [51-53].

## Conclusions

Although 'polymorphic' CNVs showed some purifying effects at miRNA *loci*, there were still many miRNA *loci *that overlapped with known CNV regions (Additional file 2 and Table 2), including those that are highly validated and confirmed by high-quality genotyping (Table 1). Taking into account the CNV genome coverage (1.2% 'polymorphic-SMC' and 2.3% 'polymorphic-DC') and the relatively small overlapping fractions (0.39 and 0.20, respectively) between the two sets of 'polymorphic' CNVs analyzed in this study, we estimated that up to 10% of the human genome is covered by highly polymorphic CNVs. This fraction corresponds to approximately 30 highly polymorphic CNV-miRNAs in the human genome (extrapolation of the fraction of miRNA *loci *covered by highly polymorphic CNVs analyzed in this study). It is likely that at least some of these *loci *are among the CNV-miRNAs identified from the top-validated 'DGV-deposited' CNVs (Table 2 and Additional file 2).

CNV-miRNAs are potential functional variants and should be considered high priority candidate variants in genotype-phenotype association studies, especially when they are located in regions implicated by linkage or association studies. As indicated in Table 1, only a small fraction of CNV-miRNAs were genotyped in three HapMap populations, which provides precise information about their polymorphisms. This is mostly due to the lack of appropriate methods for precise characterization of CNV polymorphisms. Although several genome-wide approaches that substantially fulfill the above requirement were proposed recently, a simple and inexpensive method that enables accurate characterization of several CNVs of interest in a large number of samples is still needed. The lack of such a method significantly hampers the analyses of CNVs and their correlation with the phenotype. To verify and characterize the polymorphisms of all CNV-miRNAs, we are developing several medium-throughput assays suited for large scale population studies that are focused on selected CNVs of potential functional effect. These assays will take advantage of the MLPA-based strategy proposed previously [54-56].

## Methods

Genomic coordinates (hg18) of 718 human miRNA *loci*, 13 600 093 SNPs (only annotated as 'single'), 29 133 CNVs (only annotated as 'Copy Number') and 58 *loci *implicated in microdeletion syndromes were downloaded from miRBase v13.0 http://www.mirbase.org, dbSNP build 130; Apr 30, 2009, Database of Genomic Variants update Aug 05, 2009 http://projects.tcag.ca/variation and DECIPHER database v5.0 [50]http://decipher.sanger.ac.uk, respectively. The coordinates of 1319 CNVs described as 'polymorphic-SMC' and 5037 CNVs described as 'polymorphic-DC' were extracted from supplementary materials of references [32] and [22], respectively. The number of miRNA *loci *and fraction of genome covered by CNV regions were calculated using 'feature coverage' and 'base coverage' tools available on the Galaxy, web portal for large-scale interactive data analyses [57].

The expression profiles of CNV-miRNAs were generated with the use of mimiRNA database [36] that summarizes expression data from miRNA Atlas [58], quantitative real-time PCR [59,60] as well as microarray and deep sequencing data from GEO (Gene Expression Omnibus) [61]. The assessment of evolutionary conservation of microRNAs was done based on the data available at the miRBase and blast searches of the vertebrate genomic sequences with human pre-microRNAs.

All statistical analyses were performed using Statistica (StatSoft, Tulsa, OK). The Fisher's exact test for comparison of SNPs frequency in the annotated miRNA sequences and in the total genome sequence was calculated as described in [62], with the use of the online tool available on webpage http://www.langsrud.com/fisher.htm.

## Authors' contributions

MM performed the computational analysis, literature screening, participated in the manuscript preparation. MS participated in the computational analysis (sequence conservation analysis) and the manuscript preparation. WJK participated in the design of the study and in the manuscript preparation. PK performed the statistical analysis, conceived of the study, and participated in its design and coordination. All authors have read and approved the final manuscript.

## Supplementary Material

Additional file 1 **SNPs identified in pre-miRNA sequences**. Excel table containing list of SNPs identified in annotated pre-miRNA sequences. Click here for file

Additional file 2 **miRNA identified in CNV regions**. Excel table containing list of pre-miRNA annotated sequences identified in 'DGV-deposited' CNVs. Click here for file

Additional file 3 **Expression profiles of selected CNV-miRNAs**. Expression profiles of selected CNV-miRNAs generated with the use of mimiRNA database [36]. The expression of all miRNAs was normalized in each tissue to a standard score spanning 1-1,000 (1,000 represents highest expression observed in tissue). The bars represent mean expression measured in multiple experiments and the error bars represent standard error of the mean. The variability of the expression level is indicated by colors (red - lowest variability; yellow - highest variability). Details can be found on mimiRNA webpage http://mimirna.centenary.org.au and in [36]. Click here for file

Additional file 4 **miRNAs located in CNVs with well defined breakpoints**. Excel table showing characteristics of miRNAs located in CNVs with well defined breakpoints. Click here for file

Additional file 5 **miRNAs located in chromosomal regions implicated in microdeletion/microduplication syndromes**. Excel table containing list of miRNAs located in chromosomal regions implicated in microdeletion/microduplication syndromes (DECYPHER v5.0 [50]). Click here for file
