# Supplementary material for: Copy number variation of microRNA genes in the human genome
Source: BMC Genomics. 2011 Apr 12;12:183. doi: 10.1186/1471-2164-12-183 (PMC3087710; doi:10.1186/1471-2164-12-183)

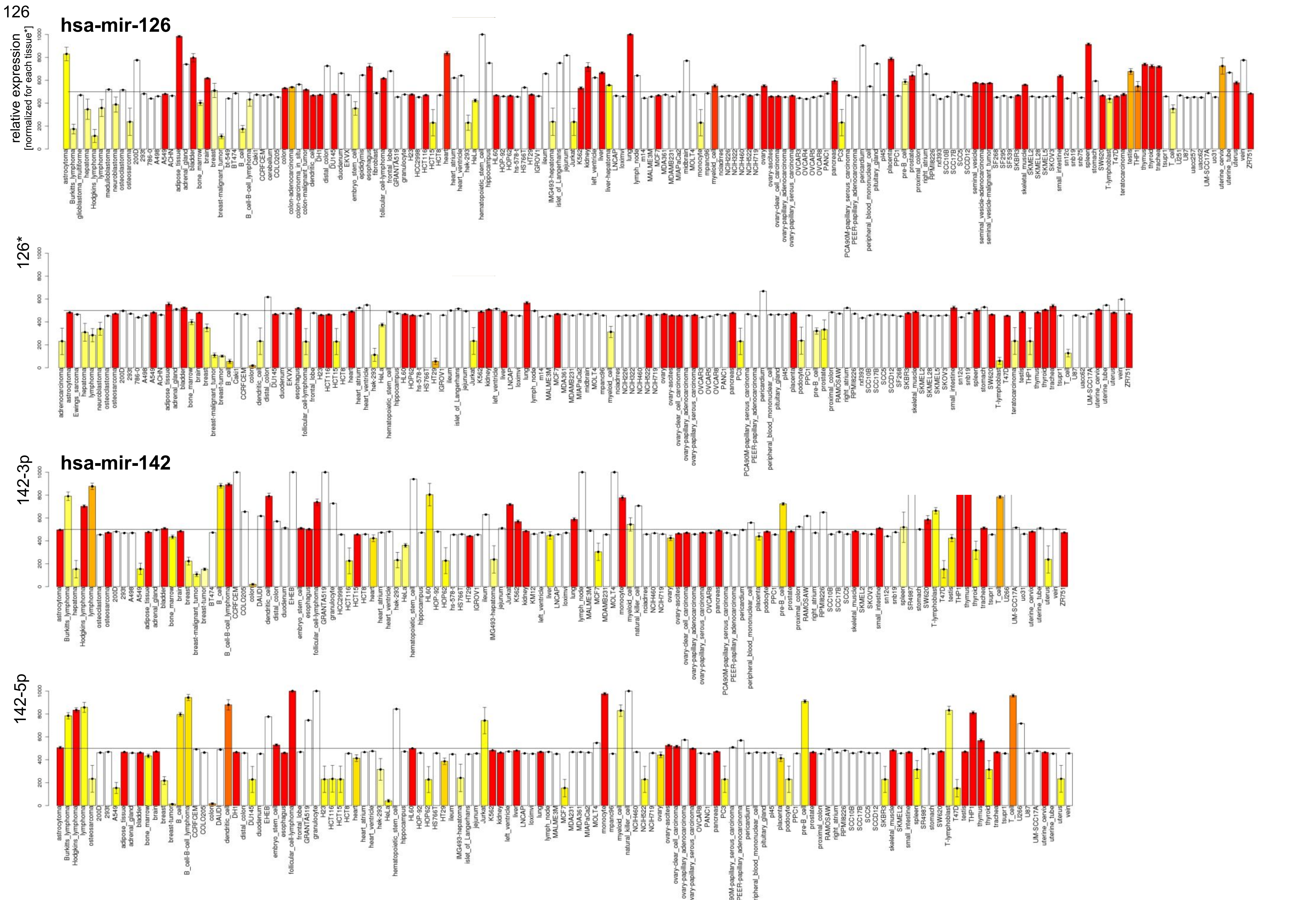

*highest expression in each tissue was adjusted to 1000 units

hsa-mir-149

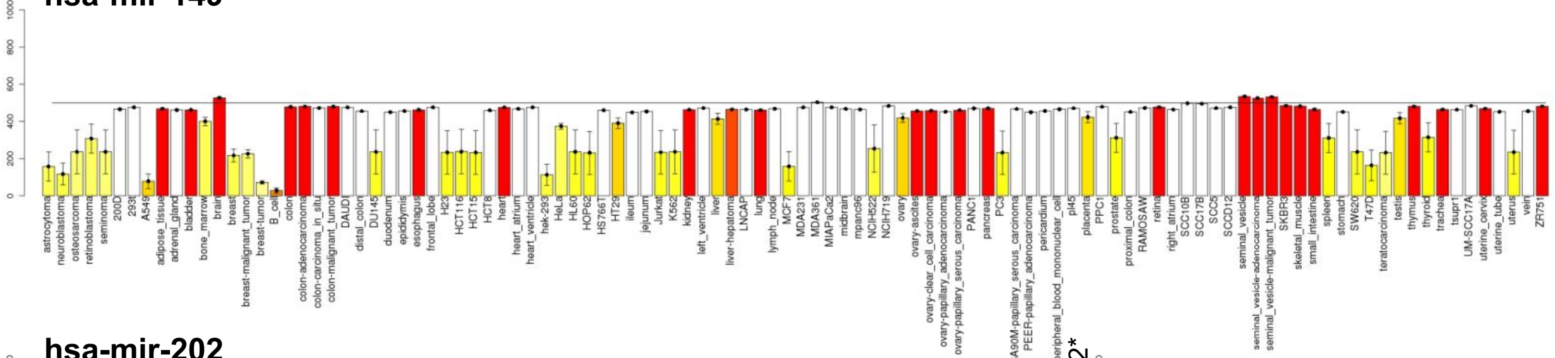

202

hsa-mir-202

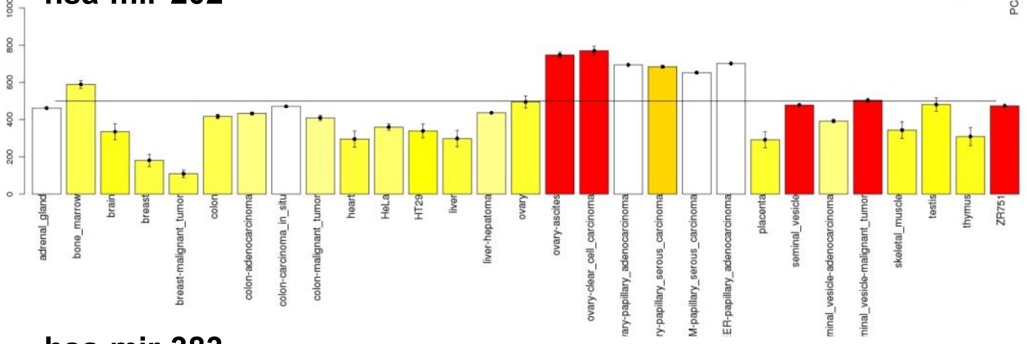

202*

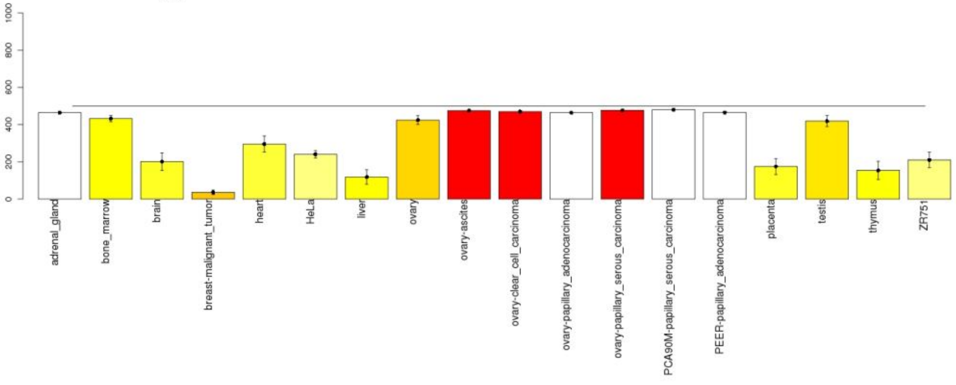

hsa-mir-383

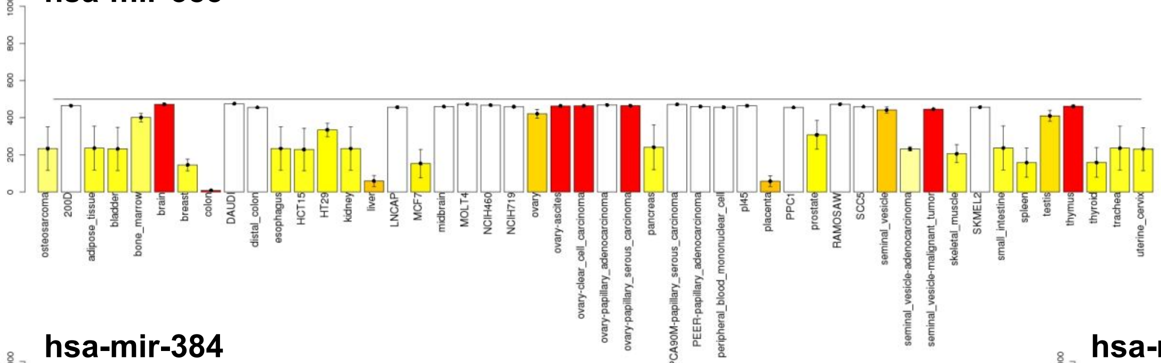

hsa-mir-384

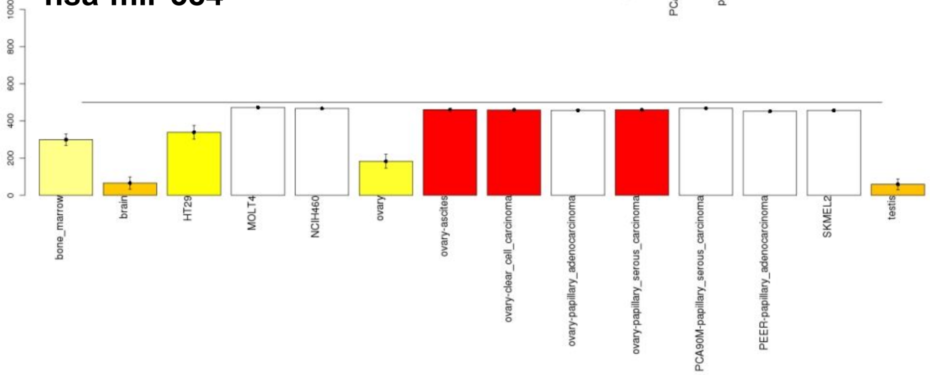

hsa-mir-514

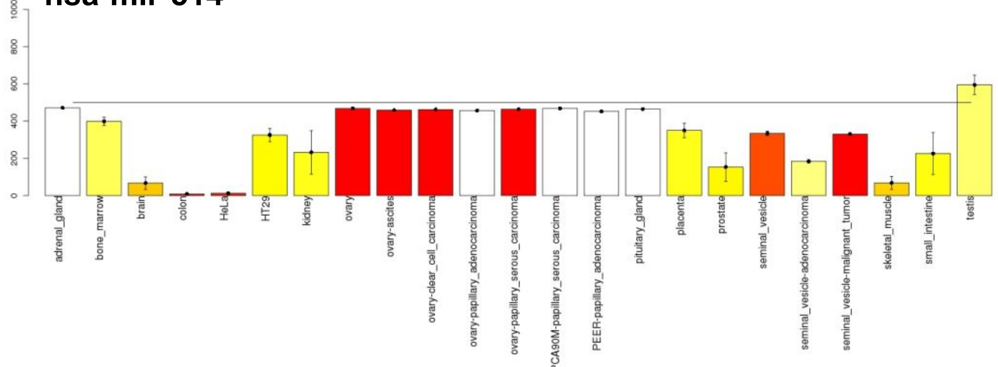

hsa-mir-566

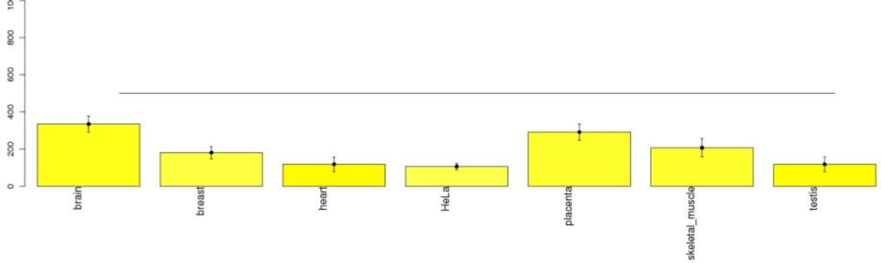

hsa-mir-570

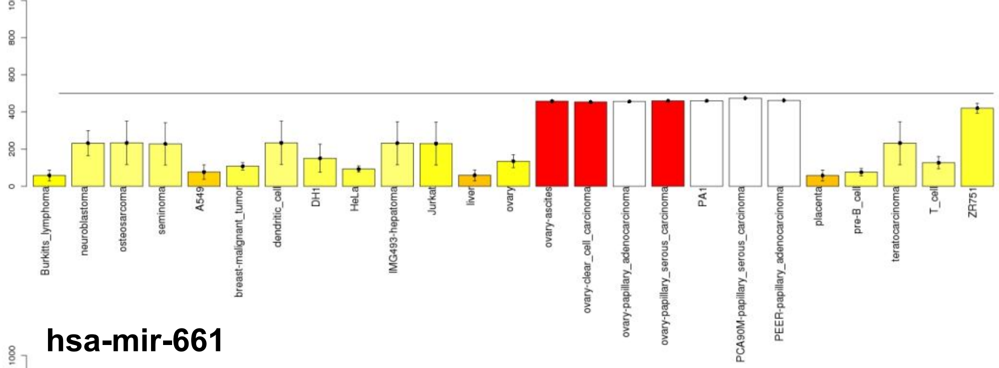

hsa-mir-650

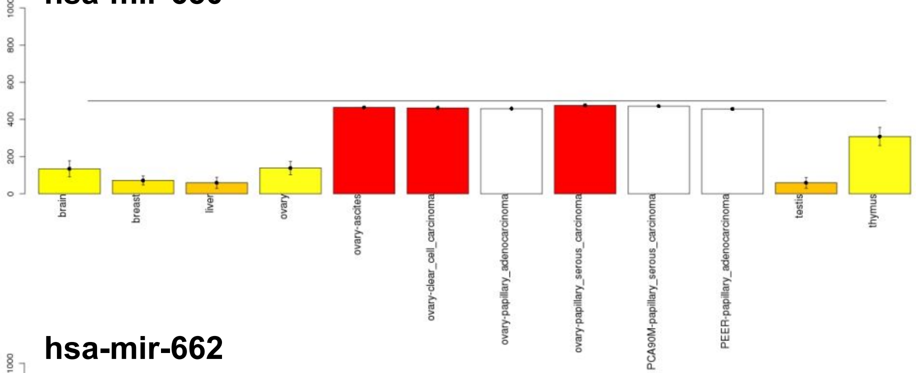

hsa-mir-661

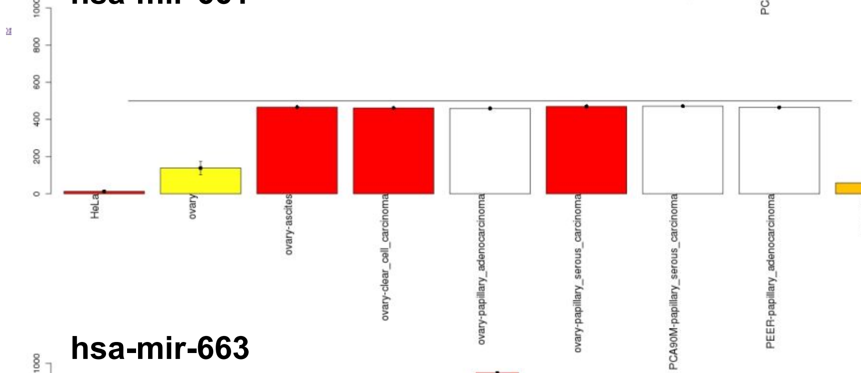

hsa-mir-662

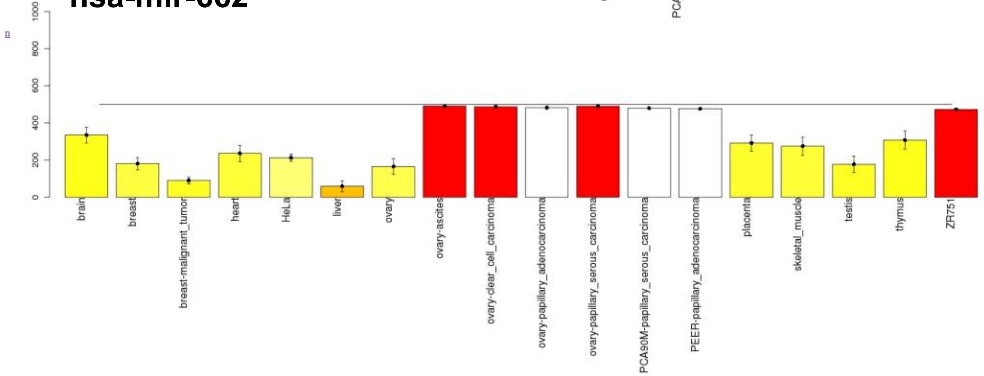

hsa-mir-663

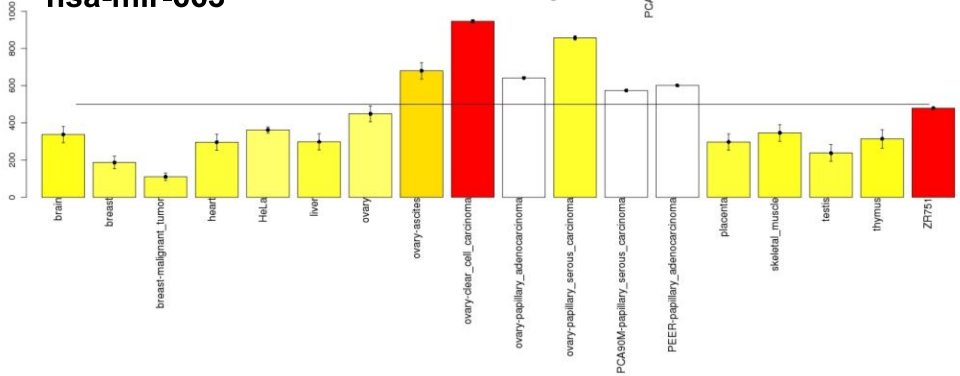

Supplement: Additional file 3 — Expression profiles of selected CNV-miRNAs. Expression profiles of selected CNV-miRNAs generated with the use of mimiRNA database [36]. The expression of all miRNAs was normalized in each tissue to a standard score spanning 1-1,000 (1,000 represents highest expression observed in tissue). The bars represent mean expression measured in multiple experiments and the error bars represent standard error of the mean. The variability of the expression level is indicated by colors (red - lowest variability; yellow - highest variability). Details can be found on mimiRNA webpage http://mimirna.centenary.org.au and in [36]. [file 1471-2164-12-183-S3.PDF]
